# *Listeria monocytogenes* Biofilms in the Wonderland of Food Industry

**DOI:** 10.3390/pathogens6030041

**Published:** 2017-09-04

**Authors:** Angelo Colagiorgi, Ilaria Bruini, Pierluigi Aldo Di Ciccio, Emanuela Zanardi, Sergio Ghidini, Adriana Ianieri

**Affiliations:** Department of Food and Drug, University of Parma, Strada del Taglio 10, Parma 43126, Italy; ilaria.bruini@studenti.unipr.it (I.B.); pierluigialdo.diciccio@nemo.unipr.it (P.A.D.C.); emanuela.zanardi@unipr.it (E.Z.); sergio.ghidini@unipr.it (S.G.); adriana.ianieri@unipr.it (A.I.)

**Keywords:** food safety, biofilms, *Listeria monocytogenes*, extracellular matrix, food industry

## Abstract

The foodborne pathogen *Listeria monocytogenes* is a concern in food safety because of its ability to form biofilm and to persist in food industry. In this mini-review, the issue represented by this pathogen and some of the latest efforts performed in order to investigate the composition of biofilms formed by *L. monocytogenes* are summarized.

## 1. Introduction

*Listeria monocytogenes* are a Gram-positive, rod-shaped ubiquitous bacteria that cause human listeriosis, a rare disease associated with high hospitalization and mortality rates—causing septicemia, meningitis, miscarriage, and stillbirth—that affects the elderly, pregnant women, newborns, and immunocompromised adults [1]. The ingestion of contaminated food (such as meat, poultry, dairy, vegetables, and ready to eat products) is the main route of transmission to humans [2].

This foodborne pathogen is well known to form biofilms, that are structured communities of bacterial cells embedded in a self-produced matrix of extracellular polymeric substances (EPSs), characterized by an altered phenotype and gene expression [3]. *L. monocytogenes* are able to form biofilm on several surfaces used in the food industry, representing a serious concern for food safety because it could serve as source of contamination. In fact, *L. monocytogenes* has been isolated from a wide range of processed foods [4,5,6,7] and also cooked food can be contaminated as the results of post-process contamination. In this mini-review, we summarized some of the efforts that have been conducted in order to characterize biofilms of *Listeria monocytogenes*.

## 2. *L. monocytogenes* in the Food Industry

*L. monocytogenes* is able to attach to many food-contact surfaces, such as stainless steel, polystyrene and glass [8]. It has been found to persist even for several years in food industries, where it could cause recurrent cross-contamination of food products [9]. In this field, Whole Genome Sequencing (WGS) is an important tool. In the recent years it has emerged as the best method to perform epidemiological surveillance and investigation of outbreaks, since it allows a more sensitive differentiation of bacterial subtypes respect to other classical molecular subtyping methods [10,11]. Very detailed information about the phylogenetic relationship results from the analysis of Single Nucleotide Polymorphisms (SNPs) in the whole genome of isolated strains. From 2013, the U.S.A. started to sequence all the *Listeria monocytogenes* isolates collected from clinical, food, and environmental sources (as part of the GenomeTrakr project) [12], since WGS was able to differentiate strains of *L. monocytogenes* isolated from the same outbreak that showed paucity of genetic diversity [13,14,15]. For this reason, this tool could be applied to study persistence of listerial strains in the food industry—e.g., by tracking the source of contamination—and to investigate the molecular mechanisms linked to persistence. There are different theories trying to explain the persistence of *L. monocytogenes* within food processing plants. One theory concerns the presence of particularly persistent, dormant, non-dividing cells, that present an increased ability to survive environmental stresses [16,17]. According to Carpentier and Cerf [18], the persistence could be related to the inability to remove cells from niches (hard to clean sites) within the food environment, where they can survive and grow, rather than the presence of strains with unique properties leading to persistence. On the contrary, other authors argue that bacterial persistence is more likely related to biofilm formation, since cells within biofilms are more resistant to biocides and stress conditions (including cleaning and disinfection/sanitization) [19,20,21].

**Figure 1:**
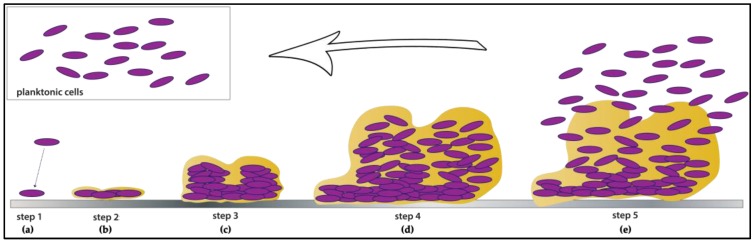
Schematic representation of the biofilm development stages. (**a**) The first step involves planktonic cells reversible attachment to surfaces; (**b**) the adhered cells begin to form a monolayer and to produce extracellular matrix; (**c**) the cells within the self-produced extrapolymeric matrix continue to grow and form multilayered microcolonies; (**d**) cells are irreversibly attached to the surface and embedded in the matrix: the biofilm is mature; (**e**) in the last stage of biofilm formation, cells are able to detach from the biofilm and to return in planktonic form, ready to colonize new surfaces. Adapted from [24].

Microbial cells within a biofilm are organized in complex structures in which they are embedded in a self-produced matrix of extracellular polymeric substances (EPSs), that are responsible for the adhesion to surfaces and cohesion in the biofilm [22]. EPSs also confer several features to biofilm, such as the structure complexity, a higher resistance to removal and destruction, and an increased resistance to antimicrobials. Furthermore, in the last stage of biofilm development, microbial cells are able to detach from the biofilm and to disperse into the environment (in their planktonic form), representing a potential source of contamination [23] (Figure 1).

Some important potential sources of contamination by *L. monocytogenes* are represented by environmental surfaces such as walls and floor: people, air, and cleaning systems may serve as vectors for microorganisms transmission to food [25]. Floors, waste water pipes, bends in pipes, conveyor belts, rubber seals, and stainless steel surfaces are some of the most common sources involved in biofilm accumulation, as well as improperly cleaned and sanitized equipment and airborne microbiota, that are also involved in contamination [25].

Several studies reported a minor sensitivity to biocides of *L. monocytogenes* within biofilm respect to its planktonic counterpart [26,27,28,29,30] and that the ability of bacteria to form biofilm enhances the resistance of this microorganism to antimicrobials [29,31]. Many antimicrobials that are used in food industry for removing *L. monocytogenes* are usually able to reduce and inactivate the microorganisms. Nonetheless, there are still some risks related to the detachment and the regrowth of the cells [32,33]. For all these reasons, biofilms still represent a great concern in food industry.

## 3. Biofilms by *L. monocytogenes*

Biofilm formation by *L. monocytogenes* is influenced by a multitude of conditions, hereafter briefly summarized. Temperature plays an important role in favoring biofilm formation [8,34], as well as the nature of adhesion surface and its hydrophobicity [35]. Most of the studies focused on biofilm formation of *L. monocytogenes* were conducted at temperatures higher than the low temperatures typical of food processing environments. Biofilm formation was often tested at 37 ± 2 °C, the listerial optimum growth temperature, and 25 °C (the temperature that the microorganism presents flagella). Di Bonaventura et al. [8] analyzed biofilm formation of 44 different isolates of *L. monocytogenes* on different surfaces at four temperatures (4, 12, 22, and 37 °C). They observed complex organization of *L. monocytogenes* biofilms at both 22 and 37 °C in terms of cell number and EPSs produced, whereas a rudimentary biofilm consisting of sparse cluster of cells and few EPSs were observed at both 4 and 12 °C. Authors suggested that these results were not due to a different cellular physiology but rather to a reduced growth of bacteria.

According to this work [8], *L. monocytogenes* was able to form biofilms at 4 and 12 °C with higher levels on glass compared to the more hydrophobic stainless steel and polystyrene. Nonetheless, authors also observed a significantly higher biofilm production at 37 °C than at 4 °C. Similar results were obtained by Tomičić et al. [36]. They observed a significantly stimulated biofilm formation of *L. monocytogenes* strains at 25, 37, and 42 °C in comparison to the lowest incubation temperature 7 °C. Also Chavant et al. [37] observed the ability of *L. monocytogenes* LO28 strain to colonize a polytetrafluoroethylene (PTFE) surface at 37 °C, but not at 8 °C.

On the other hand, other authors observed biofilm formation at refrigerator-like temperatures, such as Bonsaglia et al. [38], that observed biofilm formation at 4 °C on different surfaces, with higher levels of biofilm on stainless steel and glass compared to polystyrene. Also Norwood and Gilmour [39] reported two *L. monocytogenes* isolates that were able to adhere in the same way at 4 °C and 30 °C.

It is possible that biofilm formation at low temperatures could be regulated by genes that are not implicated in this process at higher temperatures, as recently reported by Piercey et al. [40]. They observed enhanced biofilm formation at 15 °C in bacteria mutated in 9 genes not previously linked to this process, as well as in other bacteria carrying mutation in 10 genes known to be involved in biofilm formation at higher temperatures.

The ability of *L. monocytogenes* to produce biofilms at low temperatures used during food processing and storing increases the likelihood of cross-contamination.

A strong strain-to-strain variation in biofilm forming ability was observed. Although some authors reported a correlation between lineages and biofilm forming ability (with lineage II strains presenting higher levels of biofilm production) [41], other results did not support these findings [8].

Biofilm architectures were also observed to be different among strains. Two recent studies analyzed the three-dimensional structure of several strains of *L. monocytogenes* by Confocal Laser Scanning Microscopy [23,42]. In both the articles, different structures were observed that were different in biovolume, mean thickness, and roughness, ranging from flat multilayers to complex honeycomb-like structures. Among the observed structures, the honeycomb-like morphotype was the dominant one, characterized by layers of cohesive cells, heterogeneously distributed, decorated with hollow voids and localized pockets containing dead cells and extracellular DNA (eDNA) [42].

In natural environments, such as in food industry, biofilms are composed by multiple bacterial species (mixed-species biofilms). Mixed-species biofilms have been found to be more resistant to disinfectants and sanitizers than the rarely occurring, more studied, mono-species biofilms.

Biofilm formation of *L. monocytogenes* in mixed-species biofilms was also studied. In a study of 2004, the effect of 29 food-related bacterial strains on *L. monocytogenes* biofilm formation on stainless steel was tested [43]. It was observed that co-cultivation with four of the tested strains was able to increase listerial biofilm formation, whereas the other strains showed no effect or resulted in decrease of biofilm formation. Other studies showed either an increase of number of listerial cells within biofilms when it was co-cultivated with *Flavobacterium* spp. [44], or a similarity in the number of cells between mono-species listerial biofilms and mixed-species biofilms in co-cultivation with different *Staphylococcus aureus* strains [45]. In contrast, different studies about co-cultivation of *L. monocytogenes* with food-related strains showed that some species—such as *Staphylococcus sciuri* [46], *Pseudomonas fragi* [39], *Enterococcus durans*, and *Lactococcus lactis* [47]—were able to prevent the increase of listerial population within biofilms.

The influence of other factors on mono-species and multi-species listerial biofilms was also investigated by Silva et al. [48] that analyzed the formation of *L. monocytogenes, Enterococcus faecium*, and *Enterococcus faecalis* (that were found to be present in cheese processing plants in previous studies) mixed-species biofilms on stainless steel at various temperatures and contact times. They observed that the presence of *Enterococcus* spp. and temperatures affected the growth of *L. monocytogenes*; in particular, at 25 °C, *L. monocytogenes* biofilm growth was higher in mixed-species cultures, whereas the opposite effect was observed at 39 °C.

The resistance to antimicrobials of *L. monocytogenes* mixed-species biofilms was also investigated. A study by van der Veen et al. [31] revealed a higher resistance to the disinfectants benzalkonium chloride and peracetic acid of mixed-species biofilms of *L. monocytogenes* and *Lactobacillus plantarum* compared to the mono-species listerial biofilms. On the contrary, other authors did not observe an influence of culture conditions on *L. monocytogenes* resistance to antimicrobials when it was co-cultivated with *Pseudomonas putida* [49] or with *Salmonella enterica* [50].

## 4. *L. monocytogenes* Biofilm Matrix

The extracellular matrix is an important component of *L. monocytogenes* biofilms [51]. Exopolysaccharides, proteins, and eDNA are the main molecules composing the biofilm matrix of several bacteria [52]. Studies focused on characterizing the listerial biofilms showed the presence of all these structures. In particular, teichoic acids were observed to represent the major polysaccharides, since NMR analysis as well as mutational studies showed the presence of TAs in biofilms and their role in biofilm formation [53,54].

Differently from biofilms formed by other microorganisms (such as mucoid strains of *Pseudomonas aeruginosa*) in which polysaccharides are the major EPSs [55], quantification of extracellular components of listerial biofilms by Combouse et al. [56] indicated proteins as being most abundant among extracellular substances. Other authors also observed that protease treatment of biofilms was able to impair biofilm development or to induce dispersal of the cells [57,58], indicating a role of proteins in biofilm development and maintenance. Both extracellular and surface proteins (such as internalin A—inlA, and biofilm associated protein BapL) were found to be part of extracellular matrix [59]. Very recently, another class of internalin proteins (internalin L—inlL) was found to be involved in both initial bacterial adhesion and sessile development in *L. monocytogenes* EGD-e [60].

Together with polysaccharides and proteins, extracellular DNA (eDNA) was also found within listerial biofilms and demonstrated to serve as a structural component of the EPSs matrix of *L. monocytogenes* EGD-e [58,61]. eDNA has not only a structural role, since it serves also as an energy and nutrition source [52,62].

## 5. Physiological Changes in *L. monocytogenes* Biofilms

It is typical to find structural, chemical, and biological heterogeneity in a biofilm, resulting from the presence of gradients of nutrients, oxygen, and other molecules. Cells within a biofilm are therefore in a wide range of physiological states, since they are not exposed to the same conditions, and are subjected to adaptation to local environmental conditions, altered gene expression, and genotypic variation related to mutation and selection [63]. The ability of cells within a biofilm consortium to resist and persist in a variety of environments is therefore related not only to the extracellular polymeric substances composing the matrix, but also to physiological changes in the cells.

Different studies have investigated the physiological changes of *L. monocytogenes* cells within a biofilm [64,65,66,67]. It was recently observed that the composition of the bacterial membrane is subjected to changes, such as a different content in fatty acids of cells within a biofilm respect to their planktonic counterpart [64]. In particular, Dubois-Brissonet et al. [64] observed the content of saturated fatty acids (SFA) were 12.7% higher (*p* < 0.05) in biofilm cells than in planktonic ones, whereas the content of branched-chain fatty acids (BCFA) was observed to be significantly lower. The increase in SFA and decrease in BCFA was already observed in *L. monocytogenes* on simply adhered cells [65], and is known to lead to a higher phase transition temperature, density of packing, and bilayer stability [66]. These physiological changes in biofilms allow adaption to stressful conditions and enhancement of bacterial survival and resistance to antimicrobial agents [64]. Also Miladi et al. [67] observed a change in fatty acids content of bacterial membrane after starvation and freeze stress exposure (−20 °C) of *L. monocytogenes* cells, shifting from a prevalence of iso- and anteiso-branched FAs having 15 and 17 carbon atoms, to the presence of a high proportion of straight FAs such as long-chain hexadecanoic acid (16:0), heptadecanoic acid (17:0), and octadecanoic acid (18:0). Furthermore, they observed an increase in biofilm production and in surface hydrophobicity (known to be involved in adhesion ability), a reduction in cell size, and a change in the shape of bacteria from rod to coccoid. In particular, a prevalence of straight FAs was found in adhered cells, suggesting a functional role of this physiological trait to the state of adhered cells. These results lead the authors to hypothesize that the virulence of *L. monocytogenes* could be enhanced by freeze and starvation stresses that cause an increase of adhesion ability. These findings are in contrast with other studies that observed a reduced biofilm forming ability in cold, refrigerator-like conditions, as already reported in Section 3. For this reason, the effect of the incubation temperature on biofilm formation by *L. monocytogenes* is certainly a topic that should be further investigated.

## 6. Conclusions and Perspectives

Biofilm formation by foodborne pathogens represents a serious concern in food industry. *L. monocytogenes* is able to form biofilms and to persist for long time, favoring contamination of foods [9].

Important contributions in the field of persistence of *L. monocytogenes* in food industry comes from the Whole Genome Sequencing studies. The GenomeTrakr project allows the genomic sequences and corresponding collection information for the samples to be publicly available via the NCBI website. This could enable the detection of more clusters (possible outbreaks) of *Listeria* infections, the identification of unrecognized sources of the bacteria, and better control of outbreaks, giving the possibility to stop them while they are still small.

Effective strategies for biofilm prevention or eradication should be applied in order to control the presence and the formation of such resistant form of pathogens. In order to reach these results, a deeper knowledge of biofilm composition is required.

Different studies have been conducted in order to better characterize the listerial biofilm extracellular matrix that represent the major component of a biofilm. These studies contributed to expanding the knowledge about biofilms formed by *L. monocytogenes*, as well as to determine the complexity and the function of the characterized components (such as TAs, exopolysaccharides, surface-associated proteins, and eDNA) during biofilm formation and development. By the way, further studies focused on characterizing the so-called “dark matter” of listerial biofilms could better help to prevent biofilm formation or to eradicate mature biofilms in the food industry.

The development of experimental systems that effectively simulate food processing environment conditions can certainly make a great contribution to the study of biofilm formation of both *L. monocytogenes* and many other foodborne pathogens, and help in the eradication of the same.
